# Hair cortisol concentration in early pregnancy on postpartum depressive symptoms

**DOI:** 10.1192/j.eurpsy.2025.1373

**Published:** 2025-08-26

**Authors:** M. Sobol, A. Błachnio, I. Hryhorchuk, E. Plucińska, J. Stasiniewicz, P. Szczepaniak, M. Meisner, M. K. Sobol

**Affiliations:** 1 Uniwersytet Warszawski, Warszawa; 2 Katolicki Uniwersytet Lubelski Jana Pawła II, Lublin; 3 Szpital Żywiec, Żywiec; 4 Akademia Humanitas, Sosnowiec, Poland; 5 Hospital Center Châlons-En-Champagne, Châlons-En-Champagne, France

## Abstract

**Introduction:**

Knowledge of the prenatal predictors of postpartum depressive symptoms is of fundamental importance in preventing them.

**Objectives:**

This study aimed to examine whether first trimester maternal hair cortisol influenced maternal postpartum depressive symptoms.

**Methods:**

The women (*N* = 75) were tested twice: in the first trimester of pregnancy and within three months after delivery. In the first trimester, they had hair samples taken and were examined using a sociodemographic survey and questionnaires: the Edinburgh Postnatal Depression Scale (EPDS), the Perceived Stress Scale (PSS-10), and the Zimbardo Time Perspective Inventory (ZTPI). After delivery, women completed a survey about the course of delivery and their child’s health, EPDS, PSS-10, and ZTPI.

**Results:**

A multiple hierarchical regression analysis was conducted to identify the predictors of postpartum depressive symptoms. In the first step, presence of maternal chronic illness and birth length were added, since these variables were associated with postpartum stress in our study. In the second step, we added the scores on depressive symptoms (EPDS) and stress (PSS-10) in the first trimester. After including hair cortisol concentration in the third step, depressive symptoms, stress, and hair cortisol concentration in the first trimester turned out to be predictors of postpartum depressive symptoms. The results showed that higher levels of depressive symptoms and stress and a lower level of hair cortisol concentration in the first trimester were associated with higher levels of postpartum depressive symptoms. We conducted mediation analyses to test time perspective as a mediator in the relationship of hair cortisol concentration in the first trimester to postpartum depressive symptoms.The results suggested that fatalistic time perspective mediated the relationship between hair cortisol concentration in the first trimester and postpartum depressive symptoms. In other words, women with lower hair cortisol concentration in the first trimester were more likely to develop postpartum depressive symptoms, and this relationship was explained by fatalistic time perspective.

**Conclusions:**

The results of our study indicate that women with very low hair cortisol concentration in the first trimester are at greater risk for postpartum depression. These are the first data to show that low level of cortisol in the first trimester can be a predictor of maternal postpartum depressive symptoms. The results of our study suggest that examining hair samples in the first trimester of pregnancy may be a valuable method for identifying women at risk of developing postpartum depression.

**Disclosure of Interest:**

None Declared

